# Supplementary material for: Adipocyte deletion of the oxygen-sensor PHD2 sustains elevated energy expenditure at thermoneutrality
Source: Nat Commun. 2024 Aug 29;15:7483. doi: 10.1038/s41467-024-51718-7 (PMC11362468; doi:10.1038/s41467-024-51718-7)
Supplement: Supplementary file 1 — Supplementary Information [file 41467_2024_51718_MOESM1_ESM.pdf]

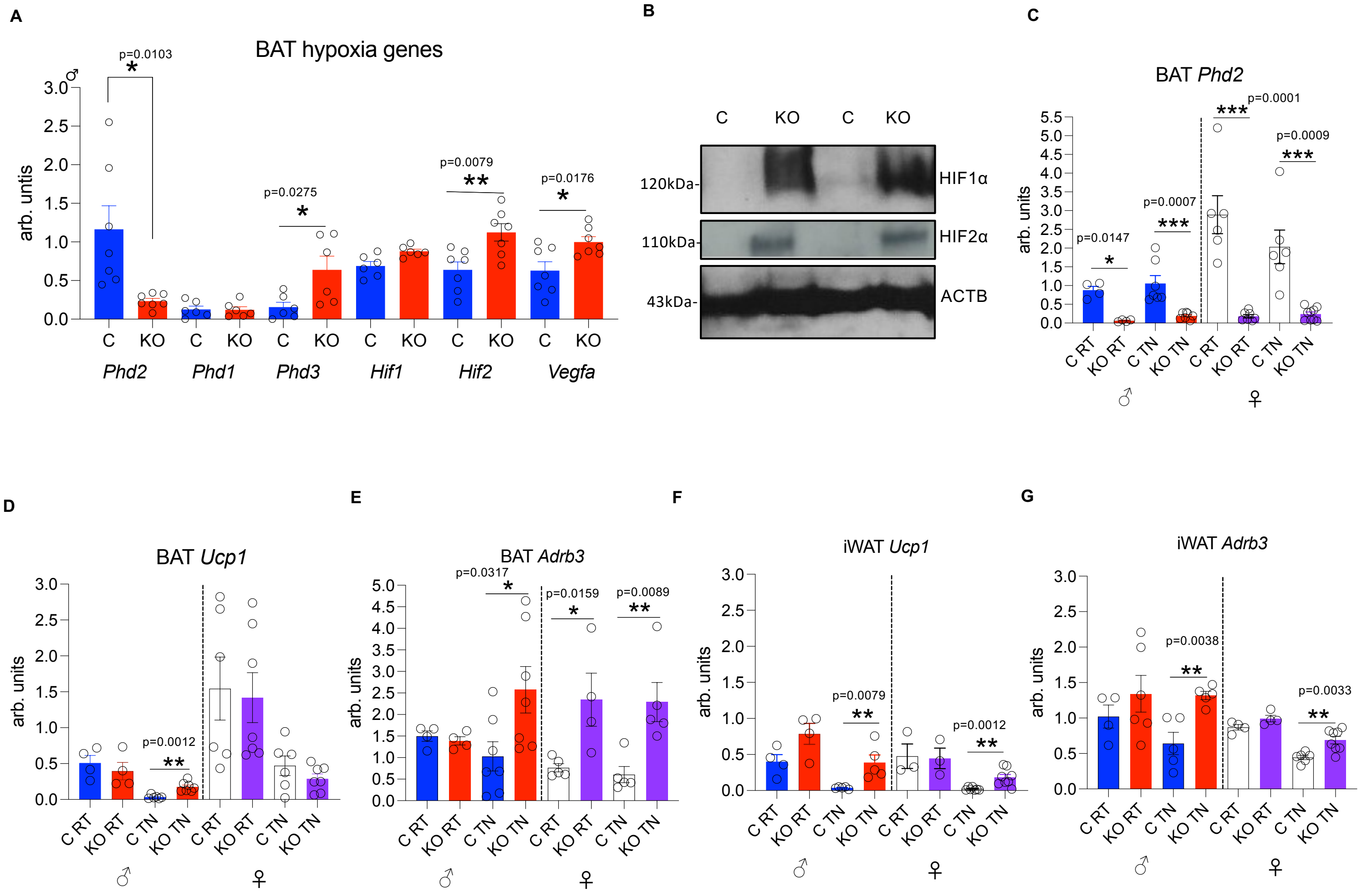

Supplemental Figure 1. *P2*KO^{ad} mice show enhanced HIF and adrenergic signaling in adipose at TN. A Efficient knock down of *Phd2* mRNA in *P2*KO^{ad} males (red) and subsequent induction of the HIF-target genes, *Phd3, Hif2 & Vegfa* (control:n=6; KO:n=7) in BAT. **B** *P2*KO^{ad} mice show stabilization of HIF1a and HIF2a isoforms in BAT. **C** *Phd2* mRNA levels in male and female mice at room temperature (RT) and thermoneutrality (TN). **D-E** Higher mRNA levels of the beta-3-adrenergic receptor, *Adrb3,* in male and female BAT and *Ucp1* in *P2*KO^{ad} male BAT. **F-G** At TN, both male and female *P2*KO^{ad} mice have higher *Adrb3 and Ucp1 levels in* iWAT. Dashed vertical line indicates not direct comparisons in males and females. Significance by Students t-test (2-tailed) or 2-way ANOVA (temperature, genotype). *p<0.05, ** p<0.01, ***p<0.001. Source data are provided as a Source Data file

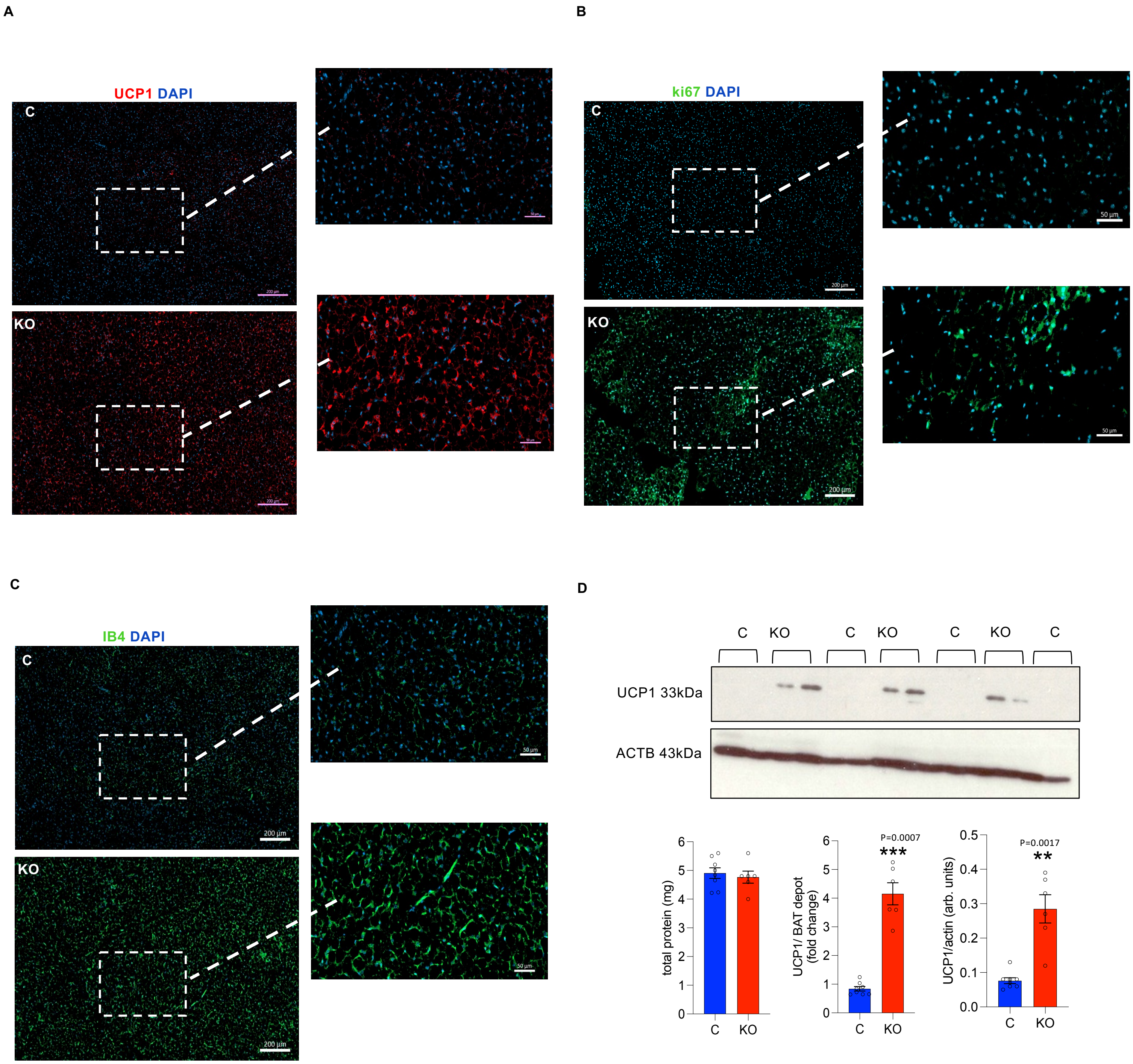

Supplemental Figure 2. Increased BAT remodeling in male *P2*KO^{ad} mice at TN.
Brown adipose immunofluorescence shows higher numbers of UCP1+ cells **A,** ki67+cells **B** and isolectin IB4 staining **C** in BAT at TN (n=3/group, biological replicates). Images (left) at 200µm and dashed square indicates image selected for higher magnification at 50µm as seen in Figure 4C; C is control, KO is *P2*KO^{ad} .Nuclei shown with DAPI in blue. **D** UCP1 protein levels in brown adipose of control (blue bars; n=8) and P2KOad (red bars, n=6) mice housed as TN. Significance by Students t-test (2-tailed) ** p<0.01, ***p<0.001. Source data are provided as a Source Data file

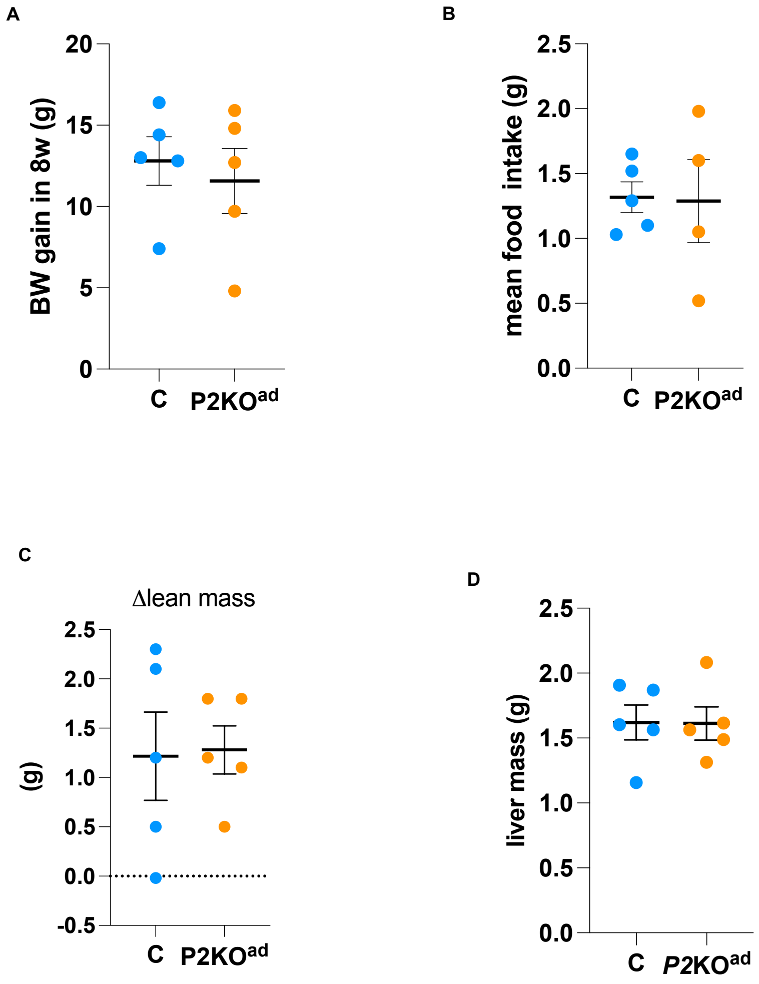

Supplemental Figure 3. Physiological and tissue responses after HFD at TN.

A Body weight gain after high fat feeding (HFD) in male control (C; light blue) and *P2*KO^{ad} (KO; orange) housed at TN (28-29^{0}C) for 8weeks (n=5/group, biological replicates). **B** Mean food intake in individually housed mice the first 3-days of HFD. **(C)** Lean mass change during HFD by TD-NMR and **(D)** liver weights corrected for body weight. Significance tested by Students t-test (2-tailed). Source data are provided as a Source Data file

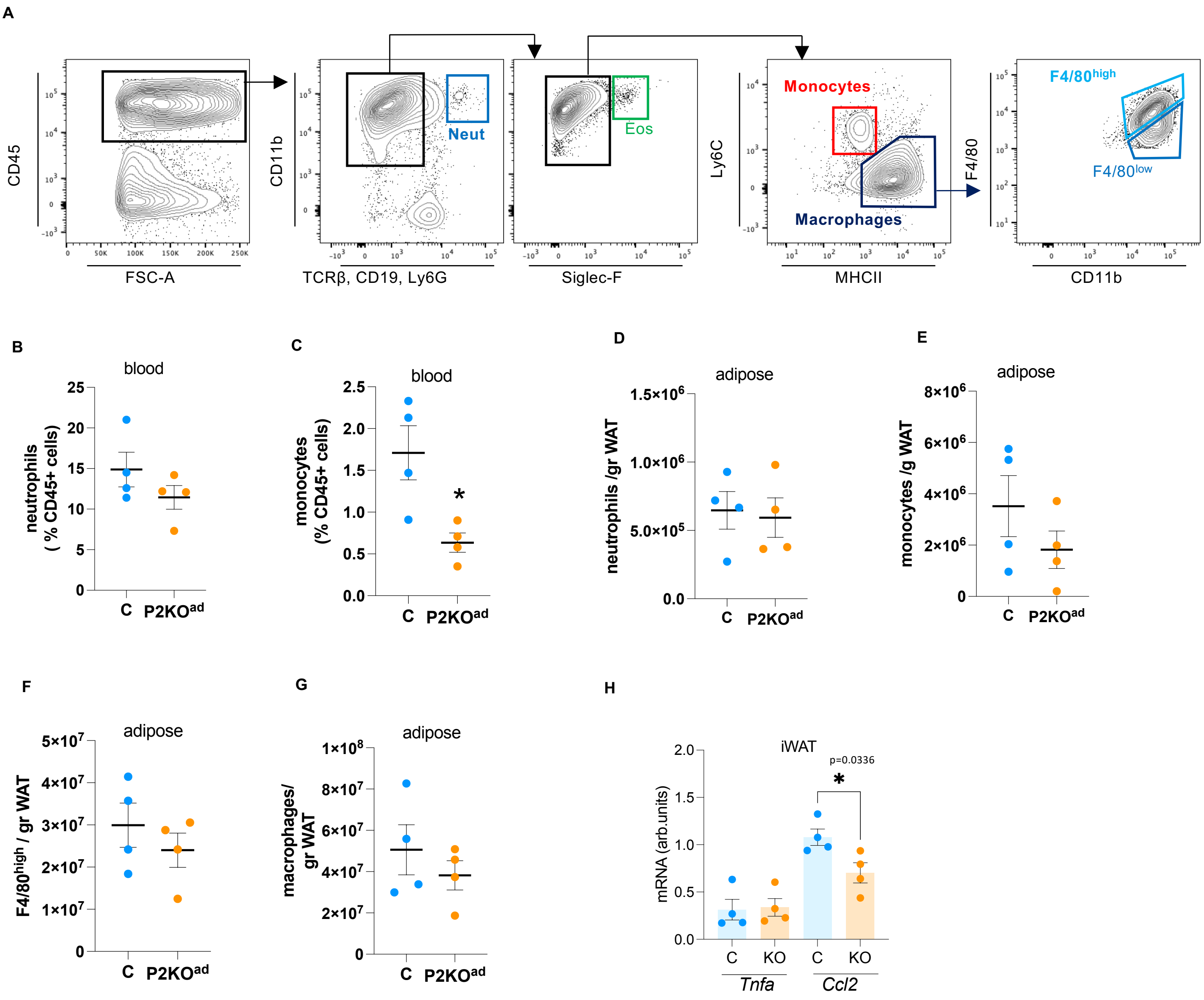

Supplemental Figure 4. *P2*KO^{ad} male mice have similar WAT inflammatory cells to control mice after diet-induced obesity at thermoneutrality.
A Representative flow-cytometric gating strategy used to identify immune cells in epididymal adipose tissue and blood from high fat fed male control (C; light blue) and *P2*KO^{ad} (KO; orange) housed at TN (28-29^{0}C), with neutrophils (Neut) identified as CD11b^{+}Ly6G^{+} cells, CD11b^{+}Siglec-F^{+} eosinophils (Eos), Ly6C^{+}MHCII^{-}Ly6G^{-}Siglec-F^{-} monocytes. Ly6C$^{-/int}$MHCII^{+} macrophages were further divided in F4/80^{high} and F4/80^{low} macrophages. **B-C** Quantification of the percentage of neutrophils and monocytes found in the CD45^{+} immune cells of the blood. **D-G** Number of neutrophils, monocytes, resident F4/80^{high} macrophages and inflammatory macrophages found per g of epididymal adipose tissue. **H** mRNA levels of Tumor necrosis factor (*Tnfa*) and Chemokine (C-C Motif) Ligand (*Ccl2*) in inguinal white adipose. n=4-5/group; Significance by Students t-test (2-tailed); n=4/group (biological replicates) *p<0.05. Source data are provided as a Source Data file

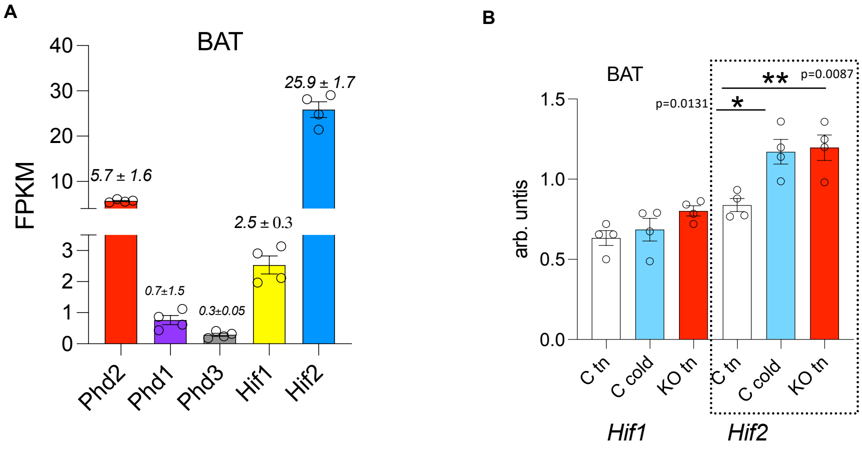

Supplemental Figure 5. Analysis of the PHD-HIF pathway components in brown adipose tissue.
A BAT RNA-sequencing data from 10-week old mice housed at RT show that *Phd2* and *HIF2a* are the most highly expressed genes (n=4/group).
B Brown adipose tissue *Hif1* mRNA levels, *in contrast to Hif2 levels*, are not regulated by acute cold exposure.
Control C57BL/6 male mice exposed to acute cold show increased *Hif2* mRNA expression levels in brown adipose tissue (light blue bars; boxed selection). Note that *P2*KO^{ad} mice (red bars, boxed selection) have similar *Hif2* mRNA levels at 29°C (tn) to control mice at cold (6°C) (n=4/group).
Significance tested with one-way ANOVA, *p<0.05, **p<0.001. Source data are provided as a Source Data file

Differentiated WT-1 cells

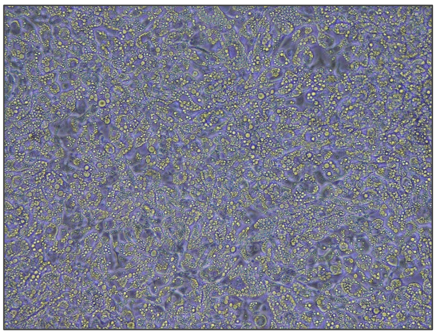

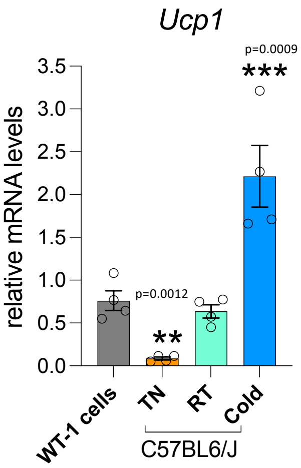

Supplemental Figure 6. **Relative *Ucp1* mRNA levels in fully differentiated WT-1 brown cells.** Comparison of *Ucp1* mRNA levels in fully differentiated WT-1 cells (image showing lipid droplets in brown adipocytes, magnification, 200μm) with levels measured in male C57BL6/J mouse brown adipose tissue housed at room temperature (RT: 21ºC), thermoneutrality (TN:29ºC) or cold (6ºC, 24h). Note that WT-1 cells have similar *Ucp1* levels to mouse BAT at RT. n=4/group; Significance by ANOVA, ***p<0.001. Source data are provided as a Source Data file

Table S1: Composition of diets

**Chow diet**<br>CRM E, Special Diets Services		**D12331 58%kcal fat and sucrose**<br>Research diets		
protein -crude protein	14.4%	protein	Casein, lactic, 30mesh	228g
Lipids-crude fat	2.7%	protein	Methionine, DL	2g
Crude ash	6%	carbohydrate	Sucrose, fine granulated	184g
fibre	4.7%	carbohydrate	Lodex 10	170g
Ca	0.73%	fat	Coconut oil, hydrogenated 101	333.5g
P	0.52%	Fat	Soyabean oil, USP	25g
Na	0.25%	mineral	S10001A	20g
Vit.A-3a672a	8000IU/kg	mineral	Calcium phosphate	20g
Vit.D3-3a671	600IU/kg	mineral	Sodium bicarbonate	10.5g
Vit.E-3a700	64IU/kg	mineral	Potassium citrate	4g
Cu-3b405	2mg/kg	vitamin	Choline bitartrate	2g
Fe-3b103	83mg/kg	vitamin	V10001C	1g
Mn-3b502	20mg/kg	dye	Red FD&C, Alum. Lake 35-42%	0.1g
Se-3b801	0.1mg/kg		Total	1000.1g
Co-3b304	0.65/kg			

Table S2: List of TaqMan Assays

**Gene mouse**	**Assay ID**
Egln1	Mm00459769_m1
Egln2	Mm00519067_m1
Egln3	Mm00472200_m1
Hif1a	Mm01283757_m1
Epas1	Mm00438712_m1
Vegfa	Mm00437306_m1
Ucp1	Mm01244861_m1
Adbr3	Mm02601819_g1
Tnfa	Mm00443258_m1
Ccl2	Mm00441242_m1
Ppia	Mm02342430_g1
**Gene Human**	
UCP1	Hs01084772_m1
PPIA	Hs04194521_s1
ADBR2	Hs00240532_s1

Table S3: List of primers

**Letter indicates site on *mUCP1* as per schematic in Figure 7D**	**5' Primer**	**3' Primer**
A	gttgtaggaccctccactgc	gtctgggatgaaccggagac
B	aaccatgcctggaggattcc	gctgtcctggagcttgttct
C	gaggctagacgagggcattg	aaagatgatgcagtgttgag
D	tgggaaagtgcgatgtcaca	tttgcctccatctgctcctc
E	tgtgctcattcccacagaaagt	gctgcttgcatcatgatccc
F	cctggtccaaggctgttgaa	gagctgctagtgggacttgg
G	gaagggacgctcacctttga	atctgccaggcaagctgaaa

Table S4: List of antibodies used in the study

Antigen Name	Conjugate	Clone	Manufacturer	dilution
CD11b	PE Dazzle	M170	Biolegend	1:200
CD19	BV421	6D5	Biolegend	1:200
CD206	FITC	MCA2235FA	Miltenyi	1:200
CD45.2	BV650	104	Biolegend	1:200
F4/80	PE/Cy7	BM8	Biolegend	1:200
Ly6C	AF700	HK1.4	Biolegend	1:200
Ly6G	BV421	1A8	Biolegend	1:200
MHCII IIA/IE	APCe780	M5/114.15.2	ebiosciences	1:600
Siglec-F	PE	E50-2440	BD	1:200
TCRb	BV421	h57-597	Biolgend	1:200
