# Supplementary material for: Adipocyte deletion of the oxygen-sensor PHD2 sustains elevated energy expenditure at thermoneutrality
Source: Nat Commun. 2024 Aug 29;15:7483. doi: 10.1038/s41467-024-51718-7 (PMC11362468; doi:10.1038/s41467-024-51718-7)
Supplement: Supplementary file 3 — Reporting Summary [file 41467_2024_51718_MOESM3_ESM.pdf]

nature portfolio

Corresponding author(s): Zoi Michailidou

Last updated by author(s): Aug 9, 2024

Reporting Summary

Nature Portfolio wishes to improve the reproducibility of the work that we publish. This form provides structure for consistency and transparency in reporting. For further information on Nature Portfolio policies, see our Editorial Policies and the Editorial Policy Checklist.

Statistics

For all statistical analyses, confirm that the following items are present in the figure legend, table legend, main text, or Methods section.

n/a	Confirmed	
☐	☒	The exact sample size (*n*) for each experimental group/condition, given as a discrete number and unit of measurement
☐	☒	A statement on whether measurements were taken from distinct samples or whether the same sample was measured repeatedly
☐	☒	The statistical test(s) used AND whether they are one- or two-sided<br>*Only common tests should be described solely by name; describe more complex techniques in the Methods section.*
☐	☒	A description of all covariates tested
☐	☒	A description of any assumptions or corrections, such as tests of normality and adjustment for multiple comparisons
☐	☒	A full description of the statistical parameters including central tendency (e.g. means) or other basic estimates (e.g. regression coefficient) AND variation (e.g. standard deviation) or associated estimates of uncertainty (e.g. confidence intervals)
☐	☒	For null hypothesis testing, the test statistic (e.g. *F*, *t*, *r*) with confidence intervals, effect sizes, degrees of freedom and *P* value noted<br>*Give P values as exact values whenever suitable.*
☒	☐	For Bayesian analysis, information on the choice of priors and Markov chain Monte Carlo settings
☒	☐	For hierarchical and complex designs, identification of the appropriate level for tests and full reporting of outcomes
☒	☐	Estimates of effect sizes (e.g. Cohen's *d*, Pearson's *r*), indicating how they were calculated

Our web collection on statistics for biologists contains articles on many of the points above.

Software and code

Policy information about availability of computer code

Data collection	No software were used for data collection
Data analysis	R (4.2.1), RStudio (1.1.456) and GraphPad Prism version 8 (San Diego, California), Adiposoft plugin with ImageJ (1.53c), FlowJo 10 software (Tree Star).<br>RNA-sequencing data processing and analysis, FASTQC v0.11.8 was used for quality assessment and trim galore v0.6.4 was applied for filtering adapter sequences and small reads. Clean reads were mapped to a reference genome (Genome Reference Consortium Mouse Build 38 mm10) using the alignment tools HISAT2 v2.1.0 and Bowtie2 v2.3.5.1 Expression levels were quantified Cufflinks v.2.2.1.

For manuscripts utilizing custom algorithms or software that are central to the research but not yet described in published literature, software must be made available to editors and reviewers. We strongly encourage code deposition in a community repository (e.g. GitHub). See the Nature Portfolio guidelines for submitting code & software for further information.

Data

Policy information about availability of data

All manuscripts must include a data availability statement. This statement should provide the following information, where applicable:

- Accession codes, unique identifiers, or web links for publicly available datasets
- A description of any restrictions on data availability
- For clinical datasets or third party data, please ensure that the statement adheres to our policy

The data that support this study are available in the Source data file. Un-cropped western blot images are available also in the Source data file and in Supplemental material. The custom-design proteomics SOMAscan is available through a collaboration agreement with the Novartis Institutes for BioMedical Research (lori.jennings@novartis.com). Data from the AGES Reykjavik study are available through collaboration (AGES_data_request@hjarta.is) under a data usage

agreement with the IHA. All access to data is controlled via the use of a subject-signed informed consent authorization. The time it takes to respond to requests varies depending on their nature and circumstances of the request, but it will not exceed 14 working days. Summary statistics data for each protein's genetic determinants, i.e., protein quantitative trait loci (QTLs), have been released to a public repository (GWAS catalogue), with accession numbers detailed in Gudjonsson et al. (PMID: 35078996). Mass spectrometry data (DDA or MRM) were deposited to the ProteomeXchange Consortium with the dataset identifiers PXD008819 to PXD008823, as well as the dataset identifier PASS01145, to determine the specificity of aptamers binding to target proteins (PMID: 30072576). The RNA-sequencing raw data have been uploaded to NCBIs' Gene Expression Omnibus (GEO) database and can be downloaded with GEO accession number GSE269003. Source data are provided with this paper.

Field-specific reporting

Please select the one below that is the best fit for your research. If you are not sure, read the appropriate sections before making your selection.

☒ Life sciences ☐ Behavioural & social sciences ☐ Ecological, evolutionary & environmental sciences

For a reference copy of the document with all sections, see nature.com/documents/nr-reporting-summary-flat.pdf

Life sciences study design

All studies must disclose on these points even when the disclosure is negative.

Sample size

The AGES-RS proteomics study included all 5457 individuals from the single-center prospective population-based AGES-RS study for whom protein measurements were available as well as comprehensive genotype and phenotype information.

For the animal studies (Fig.1-2-3-4-6) sample sizes were not calculated prior to performing experiments. All the in vivo metabolic phenotyping work, included largely => n = 6/group). Importantly, in many instances, paired comparisons (before and after intervention, Fig 1. 2. 3. 5) were used which increases the statistical power. Specifically, our n numbers for in vivo experiments were, figure 1: n=13-15 or n=7, figure 2: n=5-9, figure 3: n=5-12, figure 6: n=5, Supplemental Fig1: n=4-7, Supplemental Fig5: n=5. 2 independent repetitions of in vivo experiments performed for the phenotyping at room temperature and thermoneutrality (Fig.1-2-3).

Fig 5, CL administration, we used n=3-4/group (n=3 for control and n=4 for P2KOad RER and EE data) to test the effect of CL in a paired design in vivo under thermoneutrality.T he genotype difference is clear and power calculation (https://clincalc.com/stats/samplesize.aspx) results based for RER mean+/-SD allowed us to draw appropriate statistical conclusions.

In vitro work using cell lines was repeated in 3 independent experiments.

Data exclusions

Individuals with missing genotype, phenotype information and protein measurements were excluded from the study.Extreme proteomic outliers were excluded (>4.3 SD) prior to statistical analysis.

In the mouse energy expenditure data -as reported in the Source file Fig.1, Fig.6– data points excluded (highlighted in blue in the excel file) if for example a cage was opened due to sampling that would have affected monitoring of energy expenditure. This was done across groups so no data bias.

Replication

For the animal studies, the core metabolic phenotyping in both sexes described in FIG1-2-3 was replicated in 2 independent experiments (replication was successful)

experiments using cell line replicated in 3 independent experiments

Randomization

The participants in the AGES-RS study were not randomized into experimental groups. More to the point, AGES-RS is a population-based study of survivors from the 40-year-long prospective Reykjavik study (random sample of 30,795 individuals), an epidemiologic study aimed at understanding aging in the context of gene/environment interactions by focusing on four biologic systems: vascular, neurocognitive (including sensory), musculoskeletal, and body composition/metabolism.

Animal experiments: For animal experiments, mice were randomly allocated to treatment groups. In vitro, individual wells were randomly assigned different drug treatments

Blinding

Blinding for the human AGES-RS proteomics study was not relevant as this study did not compare experimental groups.

For all animal experiments described in the manuscript mice, the operator was blind to the genotype. Blinding maintained throughout in vivo and in vitro experiments.

Reporting for specific materials, systems and methods

We require information from authors about some types of materials, experimental systems and methods used in many studies. Here, indicate whether each material, system or method listed is relevant to your study. If you are not sure if a list item applies to your research, read the appropriate section before selecting a response.

Materials & experimental systems

n/a	Involved in the study
	☒ Antibodies
	☒ Eukaryotic cell lines
☒	☐ Palaeontology and archaeology
	☒ Animals and other organisms
	☒ Human research participants
☒	☐ Clinical data
☒	☐ Dual use research of concern

Methods

n/a	Involved in the study
☒	☐ ChIP-seq
	☒ Flow cytometry
☒	☐ MRI-based neuroimaging

Antibodies

Antibodies used

beta actin, ab49900 (AC-15), lot GR257267-1, Abcam
HIF-2 alpha, NB100-122, Novus Biologicals ; lot 00-9
UCP1 PA1-24894, ThermoFisher; AB_2241459
UCP1 (ab10983, RRID:AB_2241462, Abcam)
KI67 NB500-170SS, Novus Biologicals Lot H-2
isolectin B4 (B-1205, B-1205-.5, Vector Laboratories)
HIF-1α Cayman Item No. 10006421, batch 0465803-1
anti-rabbit immunoglobulins-HRP, P0399, Lot 00053036, DAKO
flow cytometry antibodies :
CD11b PE Dazzle M170 Biolegend
CD19 BV421 6D5 Biolegend
CD206 FITC MCA2235FA Miltenyi
CD45.2 BV650 104 Biolegend
F4/80 PE/Cy7 BM8 Biolegend
Ly6C AF700 HK1.4 Biolegend
Ly6G BV421 1A8 Biolegend
MHCII IIA/IE APCe780 M5/114.15.2 ebiosciences
Siglec-F PE E50-2440 BD
TCRb BV421 h57-597 Biolgend

Validation

All antibody used have been tested and validated for their target species (mouse or human) and the application (western blotting and/or immuno-fluorescence, or flow cytometry) as stated on the website of the manufacturers.HIF2:validated by biological-genetic and orthogonal strategies. Widely used for WB, CHIP https://www.novusbio.com/products/hif-2-alpha-epas1-antibody_nb100-122#reviews-publications; UCP1: WB- https://www.thermofisher.com/antibody/product/UCP1-Antibody-Polyclonal/PA1-24894 and validated also in the manuscript using positive control exposed to cold
UCP1:WB, IF validated https://www.abcam.com/en-gb/products/primary-antibodies/ucp1-antibody-ab10983
ki67-genetic strategy validated https://www.novusbio.com/products/ki67-mki67-antibody_nb500-170
HIF-1α :tested using positve controls hypoxia exposure and iron chelators https://www.caymanchem.com/product/10006421/hif-1α-(c-term)-polyclonal-antibody
Flow cytometry, antibodies were used at a concentration per recommendation by the respective manufacturer's website, or following in-house titration, to identify highest stain index. All antibodies were validated by us using appropriate FMO/isotype controls.Validated by the company https://www.biolegend.com
https://www.miltenyibiotec.com/GB-en/products/macs-antibodies/antibody-validation.html

Eukaryotic cell lines

Policy information about cell lines

Cell line source(s)	WT-1 Mouse Brown Preadipocyte Cell Line, sigma
Authentication	we authenticated by examining morphological characteristics and lipid droplet satining under the microscope and expression of UCP1 (supplemental Fig.S6)
Mycoplasma contamination	negative for mycoplasma
Commonly misidentified lines (See ICLAC register)	not misidentified lines used

Animals and other organisms

Policy information about studies involving animals; ARRIVE guidelines recommended for reporting animal research

Laboratory animals	male and female mice, age matched, background C57BL6, 8-10weeks old
Wild animals	no wild animals used
Field-collected samples	study did not involve samples collected from the field
Ethics oversight	project licence PP5702478, appropriate PILs granted under the Home Office Scientific Procedures (Animals) Act 1983 and after full ethical review by the University of Edinburgh Biological Sciences Services.

Note that full information on the approval of the study protocol must also be provided in the manuscript.

Human research participants

Policy information about studies involving human research participants

Population characteristics	The study included 5457 Icelandic individuals from the AGES-RS, i.e. 2330 "self reported" males (mean age was 76.7 +/- 5.4 years) and 3127 "self-reported" females (mean age was 76.5 +/- 5.7 years), from the population based AGES-Reykjavik study. All AGES-RS study cohort members are European White. All participants provided written informed consent. No compensation was provided for study participation.
Recruitment	The current study did not include recruitment of study participants. The Reykjavik study originally comprised a random sample of 30,795 "self-reported" men and women born between 1907 and 1935 and living the greater Reykjavik area in 1967. In 2002, the surviving individuals were invited to participate in the AGES Reykjavik study, which concluded in 2006, with a total sample size of 5764 survivors of the Reykjavik Study Cohort. For more details, see Harris et al., Am J Epidemiol 2007, 165: 1076-1087.
Ethics oversight	The AGES-RS was approved by the NBC in Iceland (approval number VSN-00-063), and by the National Institute on Aging Intramural Institutional Review Board, and the Data Protection Authority in Iceland.

Note that full information on the approval of the study protocol must also be provided in the manuscript.

Flow Cytometry

Plots

Confirm that:

- [x] The axis labels state the marker and fluorochrome used (e.g. CD4-FITC).
- [x] The axis scales are clearly visible. Include numbers along axes only for bottom left plot of group (a 'group' is an analysis of identical markers).
- [x] All plots are contour plots with outliers or pseudocolor plots.
- [x] A numerical value for number of cells or percentage (with statistics) is provided.

Methodology

Sample preparation	Murine epididymal adipose tissue were enzymatically digested with 1mg/ml Collagenase D (Roche) for 35 minutes at 37oC in RPMI 1640 (Sigma) containing 1% Foetal Bovine Serum (FBS) (Sigma). Blood was collected in EDTA-coated microtubes and 100l of blood was stained prior to red blood cell lysis with FACS lysing solution (BD). For flow-cytometry, murine cells were stained with LIVE/DEAD (Invitrogen), blocked with mouse serum and anti-murine CD16/32 (clone 2.4G2, Biolegend) and stained for cell surface markers (Table S3). DAPI was added to the cells prior to acquisition.
Instrument	Flow-data was acquired using a BD Fortessa LS6.
Software	All Flow data were analysed using FlowJo 10 software (Tree Star).
Cell population abundance	populations were identified as described in the methods and gating strategy
Gating strategy	Dead cell were excluded, determination of single cell populations using scatter profiles and based on CD45 positivity with neutrophils (Neut) identified as CD11b+Ly6G+ cells, CD11b+Siglec-F+ eosinophils (Eos),<br>Ly6C+MHCII-Ly6G-Siglec-F- monocytes. Ly6C-/intMHCII+ macrophages were further divided in F4/80high and F4/80low macrophages. B-C Quantification of the<br>percentage of neutrophils and monocytes found in the CD45+ immune cells of the blood. D-G Number of neutrophils, monocytes, resident F4/80high macrophages and inflammatory macrophages found per g of epidydimal adipose tissue.

- [x] Tick this box to confirm that a figure exemplifying the gating strategy is provided in the Supplementary Information.
